# To stimulate or not to stimulate? A rapid systematic review of repetitive sensory stimulation for the upper-limb following stroke

**DOI:** 10.1186/s40945-020-00091-x

**Published:** 2020-11-30

**Authors:** Rachel C. Stockley, Kerry Hanna, Louise Connell

**Affiliations:** 1 Stroke Research Team, School of Nursing, Faculty of Health and Wellbeing, University of Central Lancashire, Preston, PR1 2HE UK; 2 School of Health Sciences, University of Liverpool, Liverpool, UK; 3 School of Sport and Health Sciences, Faculty of Health and Wellbeing, University of Central Lancashire, Preston, UK

**Keywords:** Sensory stimulation, Upper limb, Stroke, Nerve stimulation, Rehabilitation

## Abstract

**Background:**

Repetitive sensory stimulation (RSS) is a therapeutic approach which involves repeated electrical stimulation of the skin’s surface to improve function. This rapid systematic review aimed to describe the current evidence for repetitive sensory stimulation (RSS) in rehabilitation of the upper-limb for people who have had a stroke.

**Main text:**

Methods: Relevant studies were identified in a systematic search of electronic databases and hand-searching in February 2020. The findings of included studies were synthesized to describe: the safety of RSS, in whom and when after stroke it has been used, the doses used and its effectiveness.

**Results:**

Eight studies were included. No serious adverse events were reported. The majority of studies used RSS in participants with mild or moderate impairments and in the chronic stage after stroke. Four studies used RSS in a single treatment session, reporting significant improvements in strength and hand function. Findings from longitudinal studies showed few significant differences between control and experimental groups. Meta-analysis was not possible due to the heterogeneity of included studies.

**Conclusions:**

This review suggests that there is insufficient evidence to support the use of RSS for the upper-limb after stroke in clinical practice. However, this review highlights several clear research priorities including establishing the mechanism and in whom RSS may work, its safety and optimal treatment parameters to improve function of the upper-limb after stroke.

**Supplementary Information:**

The online version contains supplementary material available at 10.1186/s40945-020-00091-x.

## Background

Upper-limb impairments are the most common deficit after stroke and are reported by at least 70% of people after stroke [1]. Recovery of the upper-limb after stroke is problematic; whilst two-thirds of people after stroke go on to walk independently, less than 20% recover full upper-limb function and over half have not regained even basic functions of the upper-limb after several years [1, 2].

Identifying efficacious treatments for the upper-limb is vital to improve function and well-being after stroke [3]. The use of electrical stimulation for rehabilitation has been recognized as a promising therapy [4, 5] and has featured in clinical stroke guidelines [6].

Repetitive sensory stimulation (RSS) is a form of electrical simulation which aims to promote improvements in motor function [7]. It is often delivered to the skin via pads or embedded in a glove [5, 8]. This type of sensory stimulation has also been referred to as electrical somatosensory stimulation (ESS) [9, 10], repetitive peripheral sensory stimulation (RPSS) [8, 11], peripheral nerve stimulation (PNS) [12] and peripheral sensory stimulation (PSS) [13] but is distinct from functional electrical stimulation which requires provision of the electrical stimulation alongside an attempted movement [14]. It is hypothesized that RSS may promote motor function by inducing cortical plasticity [15], including both functional changes in neurons and synapses, and structural changes such as changes in synapse formation, elimination, and morphology [16].

As a practical intervention, RSS may provide several benefits if it can be shown to be safe and effective. Unlike many treatments to improve function after stroke, it can be used with those people who have severe paresis of the upper-limb and so may provide a window for rehabilitation in those who could not otherwise undergo many other forms of physical therapy. The passive nature of the RSS intervention does not necessitate constant supervision by a therapist other than assistance to don and doff the RSS apparatus. Consequently, it could deliver additional benefit to patients without significantly increasing demands on already stretched therapist’s time. However, a recent survey indicated that over half of occupational and physiotherapists rarely or never use any form of electrical stimulation for the upper-limb after stroke (*n* = 78 from 142) [17]. Therefore, there is a clear need to inform therapists about the evidence underpinning the use of RSS so that they can make well-reasoned choices when considering investing in the equipment and training RSS requires. This literature review contributes to this process by providing a structured description, with focus on the key information needed by clinicians, of the current evidence evaluating RSS for the upper-limb after stroke, specifically:

- its safety,
- in whom and when after stroke it has been used,
- the dose of RSS (including the frequency, intensity, duration and settings) used, and
- its effectiveness.

The findings of this review will highlight areas where further research is needed to underpin the evidence base in addition to informing current practice by providing a guide for therapists who might be considering using RSS.

## Methods

A rapid review methodology was chosen for fulfil the aims of this study [18]. Our approach observed the key principles of knowledge synthesis (clear review objectives, predefined inclusion/exclusion criteria, reproducible search criteria, quality assessment, systematic presentation and synthesis of results) to minimize bias but provides an understanding of in whom RSS has been used and how it was used [19, 20]. However, by only including those papers published in English, utilizing one reviewer to undertake data extraction and omitting searching of grey literature, this review is able to rapidly synthesize relevant research faster than traditional reviews (e.g. Cochrane) [19]. As research evaluating RSS for the upper-limb after stroke is developing quickly (for example, two randomized controlled trials have been published since Conforto et al., completed their systematic review [8]), rapid reviews provide an ideal method of quickly assimilating evidence that can be used to inform clinical practice [20]. This review was registered with the Joanna Briggs Institute online registry (https://joannabriggs.org/ebp/systematic_review_register). It was not suitable for registration with PROSPERO.

### Search strategy

The following databases were searched for published articles and ongoing clinical trial protocols until 24th February 2020: Medline, Amed, Cinahl, Scopus, Cochrane library, Embase, Prospero and Google Scholar in addition to hand searching bibliographies of included articles. A broad date range (from inception of each database) was used to facilitate maximum inclusion [21]. Search (MeSH) terms and their combinations are detailed in Additional file 1.

Articles were included if they described comparative randomized and non-randomized controlled trials evaluating any form of non-invasive RSS for the upper-limb in human participants who were ≥ 18 years old, had a confirmed diagnosis of any form of stroke (using recognized clinical criteria e.g. the National Institute of Health Stroke Scale, NIHSS) with a specified stage of recovery (e.g. time since stroke or hyper-acute, acute, early and late subacute and chronic period) [22] and had upper-limb paresis due to their stroke. For the purposes of this review ESS, RPSS, PNS and PSS were considered to be RSS but the different parameters of delivering RSS were described to elucidate any superiority. Studies where the effects of RSS alone or as an adjunct to usual care/therapy [23] could not be determined, and those using mixed populations/etiologies unless stroke was included and discussed separately, were excluded. Interventions that included functional electrical stimulation, neuromuscular stimulation (e.g. used to elicit a muscle contraction) and all forms of brain stimulation were also excluded.

### Analysis

One author (KH) undertook the searches, verified the title and abstracts of the studies and removed duplicates. Inclusion of the trials was made by agreement between two reviewers (KH and RS); in case of conflict a third reviewer (LC) was available to reach a final decision. Data were extracted by one author (KH) and verified (RS) on a developed and agreed data charting form. This comprised participant’s characteristics, interventions, comparisons, outcomes used, study design, methodology, findings and safety outcomes [24]. The critical appraisal skills programme (CASP) tool [25] to appraise randomized controlled trials was used to structure assessment of each study. This included assessment of random allocation, blinding, similarity at baseline, loss to follow up and similarity in treatments other than experimental interventions. The quality rating of studies was presented numerically using the Physiotherapy Evidence Database tool (PEDro). The PEDro tool is an internally consistent and valid tool which comprises 10 items [26]. A score of 6 or above (out of 10) is considered to indicate a trial of moderate to high quality [26]. As the validity of the tool’s total score has been questioned, scores on individual items were also examined to gauge the quality of specific features of each included study [27].

Items from the Template for Intervention Description and Replication (TIDieR) framework was used to extract details about the implementation of RSS in the included studies to fulfil the aims of this review (Mechanism, Who, When, What) [28]. No meta-analysis was undertaken due to the heterogeneity of outcomes used by the included studies [21] and as meta-analysis is not commensurate with the aims of a rapid review [24].

## Results

**Fig. 1:**
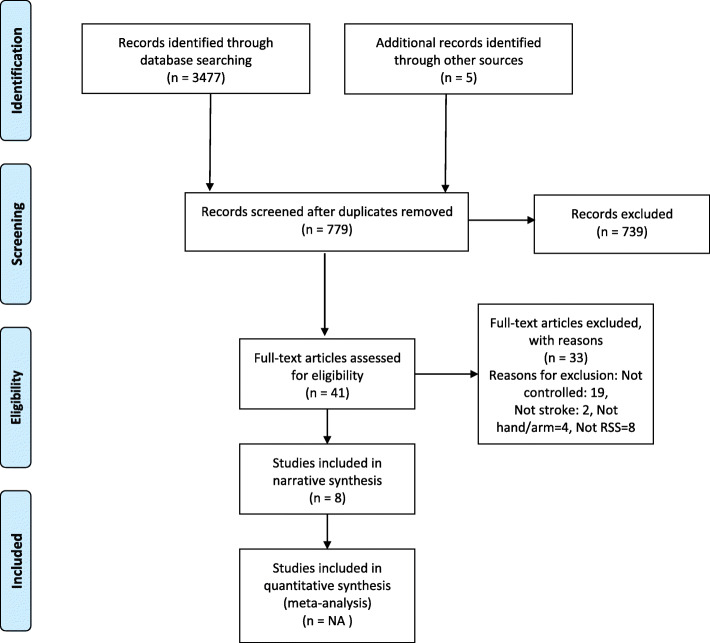
PRISMA diagram to show inclusion of articles from searches

The initial search produced eight articles that met the inclusion criteria (Fig. 1) comprising 292 participants. Six were randomised controlled trials, [9, 12, 13, 29–31] whilst two were controlled studies [11, 32]. The quality of seven from the eight included studies was considered to be moderate/high (PEDro score > 5; Table 1) whilst one was of low quality [32]. Only one of the included trials had clear methods for allocation to control or experimental groups, [13] whilst only two from the eight included studies had clear methods of therapist blinding [11, 29]. One other study did not directly compare control and experimental treatments [32].

**Table 1:** Baseline demographics of participants in included studies

Author/s, year (*n*=)	Gender (F:M)	Age (years)	Time since stroke	Baseline arm function	Stroke severity (NIHSS)	PEDro Score (/10)
Celnik et al., 2007 [12] (9)	6:3	Entire cohort: 55.2 ± 14.3	Chronic: 3.2 ± 1.6 years post stroke	JTHFT = 48 ± 9.3 FM UEA = 93.0 ± 1	–	7
Conforto et al., 2010 [11] (22)	Suprasensory group: 6:5 Subsensory group: 5:6	RSS group: 59.3 ± 1.4 Sham: 64.2 ± 3.7	Early sub-acute: RSS group: 53.1 ± 1.8 Sham: 53.5 ± 2.6 (days post stroke)	JTHFT Suprasensory: 83.3 (65–93.5) Subsensory: 90.9 (67.7–93.5) Pinch force	RSS: 4 (1–8) Sham:3 (0–9)	9
Conforto et al., 2007 [29] (11)	7:4	Entire cohort: 39.9 ± 4.2	Chronic: 4.3 ± 0.7 years post-stroke	JTHFT RSS: 89.9 (50.7–247.2) Control: 91 (40.9–215.7)	2 (1–5)	7
Conforto et al., 2002 [30] (8)	1:7	Entire cohort: 65 (38–81)	Chronic: 5 years 6 months post-stroke (range: 14 months-7 years)	Pinch force (N) RSS: 49.7 ± 8.09 N Control: 54.4 ± 10.6 N	–	7
Ghaziani, et al. 2018 [10] (102)	Low-dose group: 1:1 High-dose group: 1:1	Low-dose group: median (Q1–Q3) 71 (64–80) years High-dose group: median (Q1–Q3) 72 (64–79) years	Acute: Within 7 days post-stroke	BBT (blocks/min) Low-dose group: 10.2 ± 12.8 High-dose group: 9.3 ± 11 FMA-UE-AD Low-dose group: 33.2 ± 21 High-dose group: 33.6 ± 19.1 Hand grip strength (kg) Low-dose group: 12.0 ± 13 High-dose group: 11.0 ± 10 Palmar pinch strength (kg) Low-dose group: 1.9 ± 2.4 High-dose group:1.4 ± 2.4 Key pinch strength (kg) Low-dose group: 2.7 ± 3 High-dose group: 2.3 ± 3 Tip pinch strength (kg) Low-dose group: 1.4 ± 1.8 High-dose group: 1.3 ± 1.4	–	7
Kattenstroth et al., 2018 [31] (71)	12:34	RSS group: 64 (34–86) Sham: 59 (43–89)	Early sub-acute: 3–4 weeks post-stroke	Grip (Dynamometer) RSS: 15.55 ± 10.07 kg Sham: 32.9 ± 8.63 kg MRC: RSS: 3.73 ± 2.68 Sham: 3.98 ± 1.49 Frenchay arm test: RSS: 3.48 ± 619 Sham 3.5 ± 5.99 Wolf Motor Function test: RSS:11.76 ± 16.42 Sham: 11.56 ± 4.91	RSS group: 5.58 ± 3.22 Sham: 5.17 ± 2.23	6
Klaiput et al., 2009 [13] (10)	RSS group: 2:8 Sham: 4:6	RSS group: 63.0 ± 11 Sham: 64.4 ± 10	Early/late sub-acute: RSS group: 38.9 ± 54 days post stroke Sham: 11.9 ± 10 days post stroke	Pinch/grip (hand evaluator kit)	–	8
Peurala et al., 2002 [32] (59)	18:42	54.4 ± 10	Chronic: 3.3 (7–14) years post-stroke	MMAS: 33.7 ± 10 Limb skin sensation 11.2 ± 6	–	4

Legend: All ages and outcome scores are mean ± SD/(range) unless stated. *JTHFT* Jebsen Taylor Hand function test, *FM UE* Fugl Meyer Upper Extremity Assessment Section, *NIHSS* National institute of Health Stroke Scale, *BBT* Box and block test, *MMAS* Modified Motor Assessment Scale, *MRC* Medical Research Council Scale.

### Safety

Most of the included studies did not report safety outcomes. None of the included studies reported any serious adverse events during RSS. One study which delivered the greatest amount of RSS (up to 24 h over 4 weeks) reported mild and short lived symptoms in three participants: wrist discomfort (*n* = 1), local hyperemia (*n* = 1), and contact dermatitis (*n* = 1) [11].

### Who?

Table 1 shows the characteristics of participants in the included studies. All studies had a mix of gender and age and control and experimental groups were similar. Only three studies used the NIHSS to classify the severity of stroke [11, 29, 31]. Two of the three studies were undertaken in people after predominantly minor stroke [11, 29] (NIHSS scores < 5) whilst one study comprised people with moderate stroke severity (average NIHSS = 5.5) [31]. It was not possible to judge the severity of stroke of participants in the remaining 5 studies.

### When?

Four studies were conducted in the chronic stage of stroke (Table 1) [22]. From the others, two were in the early subacute stage [11, 31], a third was in the acute period after stroke [10] whilst another crossed two time periods (early subacute/late subacute) [13].

In chronic patients, two studies reported significantly improved performance on the Jebsen hand Function Test (JHFT) after a single RSS intervention [12, 29] whilst a third study found increased pinch grip strength immediately after RSS [30].

Three of four studies in the acute and subacute periods [11, 13, 31] reported some significant differences in impairments (grip strength, hand function and sensation) to control participants but no changes in wider function of the upper-limb. The fourth study [10] undertaken in the acute period after stroke found no differences to control participants.

### What?

#### Frequency, intensity, duration and settings

Treatment parameters are shown in Table 2.

**Table 2:** RSS treatment parameters and key findings from included articles

Author/s (year)	Total dose delivered (hours; duration x frequency)	Duration of each RSS session (hours)	Frequency of therapy	Intensity (Hz)	Format of RSS	Length of follow up	Key Findings (mean ± SD)
Celnik et al., 2007 [12]	2	2	One-off session of RSS	Each train = 5 pulses of 1-ms duration delivered at 10 Hz	2 electrode bars to stimulate ulnar and median nerves	1 h and 24 h post-test	RSS reduced JTFTH (seconds) at 1 (44.9 ± 8.2) and 24 h (43.57 ± 7.9) post-test (*p* < 0.5)
Conforto et al., 2010 [11]	24	2	3 days a week for 1 month (total of 12 sessions)	Each train = 5 pulses of 1-ms duration delivered at 10 Hz (sub/suprasensory)	2 electrode bars to stimulate median nerve	1 month and 2–3 months	RSS reduced JTHFT (seconds) 1 month: Subsensory: 49.5 ± 5.1 Suprasensory: 61.4 ± 4.9 at 1 month (*p* < 0.05) 3 months: Subsensory: 64.7 ± 5.2 Suprasensory: 54.6 ± 4.8 (ns)
Conforto et al., 2007 [29]	2	2	One-off session of RSS	1-ms duration delivered at 10 Hz	Surface electrodes to stimulate median nerve	Immediately after RSS and 1 month later	JTHFT (modified) ANOVA showed significant improvements after RSS in comparison to control with respect to the intervention (*p* < 0.001) and time (*p* < 0.0001).
Conforto et al., 2002 [30]	2	2	One-off session of RSS	Each train = 5 pulses of 1-ms duration delivered at 10 Hz	Surface electrodes to stimulate median nerve	Immediately after/24 h after RSS	Increased pinch muscle strength seen following RSS (+ 2.41 ± 0.7 N *p* = 0.017). No comparison between control and RSS group.
Ghaziani, et al. 2018 [10]	12.5	1	Daily throughout hospital stay (max 4 weeks)	Suprasensory: Continuous delivery of 10 Hz pulse width 250 μs subsensory: 10 Hz devlivered over 3 s every 2.5 min pulse width 250 μs, frequency = 10 H	Surface electrodes on wrist, elbow, and shoulder.	Post intervention and 6 months	No statistically significant differences between the sub and supra-sensory groups at any time point for any outcome measure. Box and block test (blocks/min) Subsensory, after treatment: 19.9 ± 16.2, follow up: 26.7 ± 16.7 Suprasensory: 25.9 ± 15.7, Effect size after treatment {95%CI]: − 0.8 [− 3.9, 2.3], .at follow-up: − 0.5 [− 5.2, 4.1].
Kattenstroth et al., 2018 [31]	7.5	0.75	5 days a week for 2 weeks	Each train delivered at 20 Hz burst for 1.4 s with 5 s inter-train intervals	Glove with built in electrodes contacting each finger tip	Mean 2.9 ± 1.4 days.	Significant differences in grip strength (Kg) after the intervention between sham and RSS groups. RSS: 20.2 ± 12.3 Sham: 21.3 ± 10.9 (p < 0.05)
Klaiput et al., 2009 [13]	2	2	One-off session of RSS	Each train = 5 pulses of 1-ms duration delivered at 10 Hz for 500 ms	Carbon rubberised electrodes overlaying ulnar and median nerve sites	Immediately after RSS only	Significant improvements compared to sham in lateral and tip pinch strength (pounds): Lateral: RSS: 14.1 ± 4.9 Sham: 12.9 ± 2.3 (p < 0.001) Tip: RSS: 10.8 ± 3.6 Sham: 9.8 ± 2.2 (*p* < 0.02)
Peurala et al., 2002 [32]	14.7	2 × 0.33 day	3-week inpatient period. Average 21.6 ± 6 sessions	Monophasic constant current twin pulses at 50 Hz.	Glove or shock electrode (wrist)	Unclear ?immediately after 3 week period only	MMAS - not compared to non-stimulated control: MMAS hand sensation (reported in RSS group only): Pre: 11.2 ± 6 Post: 13.7 ± 4 *p* < 0.01

Legend *JTHFT* Jebsen Taylor Hand function test, *BBT* Box and block test, *MMAS* Modified Motor Assessment Scale.

Four of the eight studies [12, 13, 29, 30] used RSS in a single treatment lasting for 2 h. These studies all used the same settings – a frequency of 10 Hz, delivered using 5 pulses of 1 ms duration (Table 2). Conforto et al. [11] also used these settings when delivering an RSS intervention for 2 h, three times a week for 1 month. Others used a range of frequencies and pulse duration as shown in Table 2.

Control interventions were matched for time and attention to blind participants, although no studies reported the effectiveness of participant blinding. Five studies [11–13, 29, 30] used a sham protocol that delivered the same duration of treatment but using a stimulus below sensory threshold. Similarly, two studies [31, 32] used a very low/no current during sham stimulation. Only one study used the same intensity of stimulation for experimental and sham interventions, but used a stimulation cycle that meant the sham group received only 2% of the active treatment provided in the experimental group [10].

### Effectiveness and dose of RSS for the upper-limb after stroke

Two studies [12, 32] found changes after RSS related to altered excitation. One observed that somatosensory evoked potentials (SEPs) became significantly more normal than those receiving placebo in 32 participants with chronic stroke after 20 min twice daily RSS treatment over 2 weeks [32]. The second reported significant reductions in intracortical inhibition after a single 2 h session of synchronous RSS for 9 people with chronic stroke compared to asynchronous and no stimulation [12]. However, one other study that measured cortico-excitability found no significant differences between two groups of early subacute stroke participants either receiving subsensory (below perceived sensory threshold, *n* = 8) or suprasensory (above perceived sensory threshold, *n* = 7) RSS for 2 h three times a week for 1 month [11]. From the four studies that delivered RSS in a single session, two found increased pinch grip strength immediately after RSS compared to the control group [13, 30]. The other two studies reported significantly improved performance on the Jebsen hand Function Test (JTHFT) after the single RSS intervention when compared to pre-test performance [12] and a sham control [29].

The findings from longitudinal studies were mixed; those that used a low-dose and/or subsensory RSS control tended to find few differences between experimental and control groups [10, 11, 31]. One study reported two significant benefits in favor of the experimental RSS intervention in hand grip and sensation, no other outcomes were significantly better than the control that received a low-dose of RSS [31]. Similarly, Ghaziani et al. (2018) found no difference between low and high doses of RSS after up to 4 weeks of daily hour long treatment [10]. Unexpectedly, after 1 month of training Conforto et al. (2010) found significant differences in favor of subsensory RSS, in comparison to suprasensory RSS, both delivered with motor training for 6 hours a week, but these difference were not maintained after 2 months [11]. Peurala et al. (2002) reported significant improvements in sensation and motor function after twice daily RSS over 3 weeks; however, whilst a sham-control group was included (who did not demonstrate significant improvements) the results between these two groups were not directly compared [32].

## Discussion

This rapid systematic review of RSS evaluated the current evidence to describe the safety of RSS interventions for the upper-limb after stroke and in whom and when after stroke it has been used. The eight included trials tended to use small samples (5 included less than 30 participants) lacked consistency in how RSS was used (frequency, duration and parameters of stimulation) in whom it was used (severity and time since stroke) and the outcomes used to indicate its effectiveness. The quality of the studies was also variable; despite many being considered to be high quality, several trials had unclear methods for allocation to control or experimental groups and therapist blinding potentially biasing their findings. Nonetheless this review provides a valuable addition to the understanding of the current evidence base underpinning RSS which can be used to inform clinical treatment decisions and clearly identifies areas for future work.

### Safety – is RSS safe?

The findings of the review indicated that there was insufficient evidence to determine the safety of RSS. Whilst most studies did not report any adverse effects, and those that were reported tended to be mild, it was not clear if and how participants were monitored for any side effects. Consequently, it should not be assumed that using RSS, particularly over long periods, will not elicit any unwanted side effects, and indicates that measurement of side effects should be clearly assessed and reported in future work.

### Patient selection – in whom could RSS work?

The heterogeneity of studies and outcome measures meant that it is not possible to draw any firm conclusions regarding the severity or when after stroke RSS should be used to have optimal benefit. Interestingly, no studies appeared to include participants with severe upper-limb deficits after stroke, despite the passive nature of RSS potentially being practically suitable for those who have had a severe stroke and cannot participate in other forms of rehabilitation that require some active movement of the limb (e.g. repetitive task practice).

Recent work has shown that arm function in the first few days after stroke can predict long term arm function for at least 75% of people after stroke [33, 34]. However, no studies included in this review stratified with respect to the participants’ potential for recovery of the upper-limb, nor undertook sub-group analysis of the effects of RSS in relation to severity. The initial severity of upper limb involvement after stroke is likely to be hugely influential to outcome [34] and the absence of overt consideration of the severity of deficit and potential for recovery may explain the equivocal findings of when and in whom RSS may work illustrated in this review. It should also be noted that several studies used RSS with other treatments considered to be usual care or therapy (e.g. motor training) [23], whilst others did not. Combining RSS with targeted motor training could enhance overall benefit by a “priming” effect of RSS upon cortical plasticity which can then be utilized by motor training [11]. This is supported by findings from a recent review of general peripheral somatosensory stimulation (which includes RSS) for the lower limb after stroke [35] which suggested that RSS should be used as an adjunct to motor training to increase the likelihood of benefit.

### Dose – how should we deliver RSS to elicit benefit?

A challenging finding of this review is that the effectiveness of RSS appeared independent of the overall amount of RSS delivered. Several studies reported significant benefits in comparison to control interventions after a single, short application of RSS, whilst longitudinal studies delivering much higher overall doses did not. Those studies that did report significant benefits after using RSS used doses ranging from 120 min to 1440 min, indicating that a linear dose-response of RSS may not exist, and even small doses of treatment may have an effect. Indeed, as the intensity (commonly considered to be a function of the duration and frequency of an intervention) of RSS required to improve upper-limb performance is not known, the assumed inactive, low-dose RSS control treatment used in several studies may, in fact, have provided sufficient stimulation to elicit similar benefits to the experimental group, accounting for the absence of significant differences [10, 11, 31]. This finding contradicts a generally accepted paradigm that more upper-limb rehabilitation leads to better outcomes after stroke and requires further investigation [36].

The studies included in this review demonstrated substantial heterogeneity in the way RSS was delivered. In contrast, a recent review of five studies (three of which were utilised in the current study) [11, 29, 30] only included RSS interventions delivered using specific treatment parameters (1 ms pulses at 1 Hz) [8]. They found a beneficial effect of RSS upon motor performance (standard mean difference: 0.67, 95% CI: 0.09–1.24) [8], suggesting that this specific mechanism of delivery of RSS are effective and potentially should be adopted in preference to unproven parameters in future studies.

Collectively the differences between results from the included studies of RSS in this review emphasise the importance of elucidating the mechanism by which an intervention is likely to work prior to evaluating effectiveness. This underpinning programme theory is vital to understand how interventions should be structured in order to be optimally effective. In particular, the findings of this review also suggest that studies exploring the dose-response of RSS are a priority for future research.

### Limitations

This review has several limitations. It was beyond the scope of this review to consider the mechanism by which RSS could elicit improvements after stroke, although others have considered this [7]. Consistent with rapid review methodology, no grey literature was searched and only studies published in English were included which is likely to introduce some bias [20]. The specific objectives of this review necessitated the exclusion of several studies as the effects of RSS could not be delineated from other interventions which were not considered to constitute usual care/therapy (e.g. constraint induced movement therapy). This meant that two trials of RSS which were included in a review in 2018 [8] and which reported findings in favor of the RSS intervention were not considered [37, 38].

## Conclusions

Repetitive sensory stimulation may be a promising treatment to improve upper-limb function after stroke but the findings of this rapid systematic review indicate that there is currently little evidence to recommend or guide its use in clinical practice.

The heterogeneity in the design and treatment parameters of trials included in this review highlights the need to prioritize the development of the theory and mechanism of action by which RSS might influence upper-limb function. The variability in how RSS was applied, and when and in whom it was used means that there is little consistent evidence on which to base its inclusion in upper-limb therapy after stroke. These shortcomings clearly identify a number of urgent priorities for future research into RSS for the upper-limb. These include elucidation of the mechanism by which and in whom it may work, development of a theoretically and empirically underpinned intervention utilizing an optimal dose of RSS and judicious selection of appropriate and robust outcome tools to capture its effects. Collectively, this work would result in future trials of the effectiveness of RSS being able to test clearly articulated causal assumptions of a theoretically and empirically informed intervention and use targeted outcome tools to produce clinically relevant findings to inform practice.

## Supplementary Information

**Additional file 1.**

## Data Availability

Data sharing is not applicable to this article as no datasets were generated or analysed during the current study.

## Abbreviations

RSS: Repetitive Sensory Stimulation
