# Supplementary material for: To stimulate or not to stimulate? A rapid systematic review of repetitive sensory stimulation for the upper-limb following stroke
Source: Arch Physiother. 2020 Nov 30;10:20. doi: 10.1186/s40945-020-00091-x (PMC7708198; doi:10.1186/s40945-020-00091-x)
Supplement: Supplementary file 1 — Additional file 1. [file 40945_2020_91_MOESM1_ESM.docx]

Appendix 1: MeSH terms used for the literature search

| Arm impairment | Stroke | Intervention |
| --- | --- | --- |
| Local sensory impairments/  Local motor impairments/  Sensory discrimination/  Range of motion/function/  Upper-limb/  Arm/  Hand/  Upper-limb paresis/  Upper-limb paralysis/  Arm paresis/  Arm paralysis/  Hand paresis/  Hand paralysis/  Hemiplegia/  Hemiparesis | Stroke/  Cerebrovascular accident/  Cerebrovascular disorder/  Cerebrovascular trauma/  Brain ischaemia/  Brain hypoxic-ischaemia/  Brain haemorrhage/  Cortical haemorrhage/  Haemorrhage/  Infarction/  Cortical infarction/  Intracranial arterial disease/  Intracranial arteriovenous malformations/  Intracranial Embolism and Thrombosis/  Intracranial haemorrhages/  Vertebral artery dissection/  Aneurysm, ruptured/ and brain/  Brain injuries/  Poststroke/  Post stroke/  Cerebrovasc$/  Cva$/  Isch?emi$ attack$/  neurologic$ deficit$ | Electric/  Electrical/  Simulation/  Nerve stimulation/  Sensory/  Afferent/  somatosensory/  Somatosensory input/  Electroacupuncture/  Repetitive/  Repetitive passive movement/ Mechanical vibration/  Transcutaneous electrical stimulation/  Peripheral nerve stimulation/  Electrodes/  Pulse/  Motor recovery/ Sensory recovery/  Sensorimotor/  Repetitive sensory/  Repetitive sensory stimulation |
| Boolean operators and combinations |  |  |
| Combined with AND/OR | Combined with AND/OR | Combined AND |
